# Heart Rate Recovery Index and Improved Diastolic Dyssynchrony in Fusion Pacing Cardiac Resynchronization Therapy

**DOI:** 10.3390/jcm13216365

**Published:** 2024-10-24

**Authors:** Andra Gurgu, Constantin-Tudor Luca, Cristina Vacarescu, Dan Gaiță, Simina Crișan, Adelina-Andreea Faur-Grigori, Alina-Ramona Cozlac, Cristina Tudoran, Mădălin-Marius Margan, Dragos Cozma

**Affiliations:** 1 Doctoral School, “Victor Babes” University of Medicine and Pharmacy, 300041 Timisoara, Romania; andra.gurgu@umft.ro (A.G.); faur.andreea@cardiologie.ro (A.-A.F.-G.); 2 Department of Cardiology, “Victor Babeș” University of Medicine and Pharmacy, 300041 Timisoara, Romania; constantin.luca@umft.ro (C.-T.L.); dan.gaita@umft.ro (D.G.); simina.crisan@umft.ro (S.C.); dragos.cozma@umft.ro (D.C.); 3 Institute of Cardiovascular Diseases Timisoara, 300310 Timisoara, Romania; alina-ramona.cozlac@umft.ro; 4 Research Center of the Institute of Cardiovascular Diseases Timisoara, 300310 Timisoara, Romania; 5 Department VII, Internal Medicine II, Discipline of Cardiology, University of Medicine and Pharmacy “Victor Babes” Timisoara, E. Murgu Square, Nr. 2, 300041 Timisoara, Romania; tudoran.cristina@umft.ro; 6 County Emergency Hospital “Pius Brinzeu”, L. Rebreanu, Nr. 156, 300723 Timisoara, Romania; 7 Center of Molecular Research in Nephrology and Vascular Disease, Faculty of the University of Medicine and Pharmacy “Victor Babes” Timisoara, E. Murgu Square, Nr. 2, 300041 Timisoara, Romania; 8 Department of Functional Sciences, Discipline of Public Health, “Victor Babeș” University of Medicine and Pharmacy, 300041 Timisoara, Romania; margan.madalin@umft.ro

**Keywords:** heart rate recovery index, diastolic dyssynchrony, cardiac resynchronization therapy, LV-only fusion pacing

## Abstract

**Background:** Restoring electrical synchrony with cardiac resynchronization therapy (CRT) reverses the heart failure phenotype developed by left-ventricular (LV) dyssynchrony. This study aimed to identify new predictors of response to LV-only fusion pacing CRT. **Methods:** A select group of patients with CRT-P indications received a right atrium (RA)/LV DDD pacing system. LV dyssynchrony was assessed via offline TDI timing focusing on the temporal difference between peak septal (E″T) and lateral wall (A“T) motion. CRT effectiveness was evaluated at each follow-up, involving the heart rate recovery index (HRRI) parameter (acceleration/deceleration time) derived from exercise testing along with the echocardiographic parameters. Patients were classified into super-responders (SR), responders (R), and non-responders (NR). **Results:** Baseline initial characteristics: 62 patients (35 male) aged 62 ± 11 y.o. with non-ischemic dilated cardiomyopathy (DCM). Ejection fraction (EF) 27 ± 5.2%; QRS 164 ± 18 ms; 29% had type III diastolic dysfunction (DD), 63% type II DD, and 8% type I DD. Average follow-up was 45 ± 19 months: 34% of patients were SR, 61% R, and 5% NR. The E″T decreased from 90 ± 20 ms to 25 ± 10 ms in SR, with a shorter deceleration time (DT) during exercise test compared to NR (109 ± 68 ms vs. 330 ± 30 ms; *p* < 0.0001). The responders present a higher HRRI (2.87 ± 1.47 vs. 0.98 ± 0.08; *p* = 0.03) compared to NR and a significantly decrease in E“T and A“T from 76 ± 13 ms to 51 ± 11 ms (*p* < 0.0001). Prolonged DT was associated with an accentuated LV dyssynchrony and nonoptimal response to CRT. **Conclusions:** The study identified new parameters for assessing responsiveness to LV-only fusion pacing CRT, which could improve candidate selection and CRT implementation.

## 1. Introduction

Dyssynchronous heart failure is associated with inefficient myocardial shortening and impaired cardiac function. Patients with QRS widths exceeding 120 ms and/or left bundle branch block (LBBB) patterns face a 15% higher mortality risk compared to their counterparts [1].

Cardiac resynchronization therapy (CRT) has the potential to restore electrical timing, yielding beneficial effects at the molecular and cellular levels, ultimately enhancing overall cardiac performance [1]. Furthermore, in non-ischemic patients with preserved atrioventricular conduction, a bicameral system utilizing right-atrial (RA) and left-ventricular (LV) leads can mitigate right-ventricular (RV) pacing-induced asynchrony often associated with biventricular pacing [2,3].

A pivotal element in CRT follow-up is ensuring the maintenance of permanent ventricular capture during exercise testing (ET). Previous studies have shown that post-exercise heart rate recovery measurement serves as a noninvasive, easily accessible tool for predicting cardiovascular morbidity and all-cause mortality [4,5]. Additionally, the calculation of the heart rate recovery index (HRRI) not only could predict the CRT response but also could provide insights into left-ventricular reverse remodeling [6].

The objective of this study was to assess the relationship between ET-derived HHRI and LV dyssynchrony in the context of CRT response and enhance candidate selection for LV-only fusion pacing CRT by identifying new predictors.

## 2. Materials and Methods

### 2.1. Inclusion and Exclusion Criteria

This study is a prospective investigation that focused on the inclusion and analysis of patients diagnosed with dilated cardiomyopathy who met the following criteria for CRT-P indication: New York Heart Association (NYHA) classes III–IV despite receiving optimal pharmacological treatment for the preceding 3 months prior to CRT, left-ventricular ejection fraction < 35%, a QRS complex duration greater than or equal to 130 ms, and sinus rhythm with preserved atrioventricular conduction.

Patients were excluded from the study if they exhibited any of the following conditions or characteristics: acute coronary syndrome or history of coronary artery disease, primary or secondary cardiomyopathies (including hypertrophic cardiomyopathy, Takotsubo syndrome, sarcoidosis, amyloidosis, or cardiomyopathy induced by toxic agents), the presence of severe comorbidities (such as renal, lung, or liver failure, cerebral insufficiency, or terminal cancer), or noncardiac conditions that limited physical activity (orthopedic conditions and neuromuscular disorders). Patients with atrial fibrillation (AF) were excluded from this study to ensure the integrity of fusion pacing. Fusion pacing relies on the synchronization between intrinsic atrial activity and ventricular pacing to optimize cardiac output. In patients with AF, irregular and uncoordinated atrial activity disrupts this synchronization, making it difficult to achieve consistent fusion pacing. Furthermore, AF often necessitates rate control strategies or rhythm management approaches that could interfere with the assessment of heart rate recovery index (HRRI) and diastolic dyssynchrony. By excluding patients with AF, we were able to focus on individuals with a stable sinus rhythm, ensuring that the effects of fusion pacing could be accurately measured without the confounding influence of atrial arrhythmias. Furthermore, patients with a CRT-D indication for primary or secondary prevention were also excluded from the study.

### 2.2. Implantation Strategy

**Figure 1:**
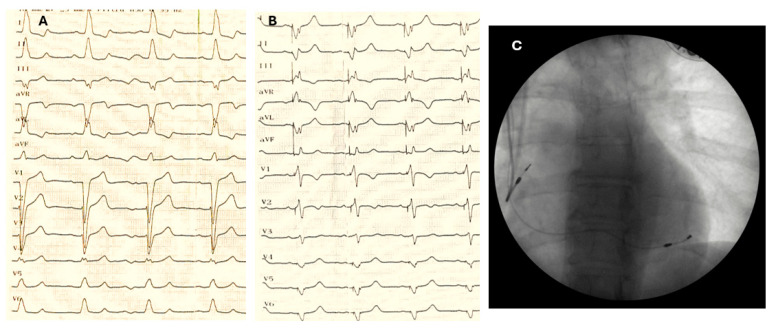
ECG before (**A**) and after (**B**) RA/LV DDD CRT; (**C**) antero-posterior fluoroscopy image.

The procedure involved direct percutaneous subclavian vein puncture to achieve permanent transvenous cardiac pacing. The initial placement of the pacing lead occurred in the right atrium, with subsequent positioning of the left-ventricular lead within different coronary sinus branches (Figure 1). Additionally, a subcutaneous pocket was created as part of the procedure.

Venography was used to find the ideal location for the implantation of LV leads. An average of 30 to 50 mL of Omnipaque (iohexol 350 mg/1 mL, GE Healthcare) was utilized for each patient as a contrast agent. Five patients (8%), all with a history of chronic renal disease, had a recorded increase in serum creatinine of ≥0.3 mg/dL 24/48 h after implantation, which was characterized as contrast-induced nephropathy. All of these individuals’ serum creatinine levels were lowered and stabilized with appropriate intravenous hydroelectrolytic rebalance and a brief cessation of potentially nephrotoxic medication. None of them required extensive nephrological procedures.

### 2.3. Echocardiography Evaluation

Transthoracic echocardiography was performed before, on the day after device implant, and at 6-month intervals, using a Vivid E9 system (GE Health Medical, Milwaukee, WI, USA) and a 2.5 or 3.5 MHz phase array transducer.

**Figure 2:**
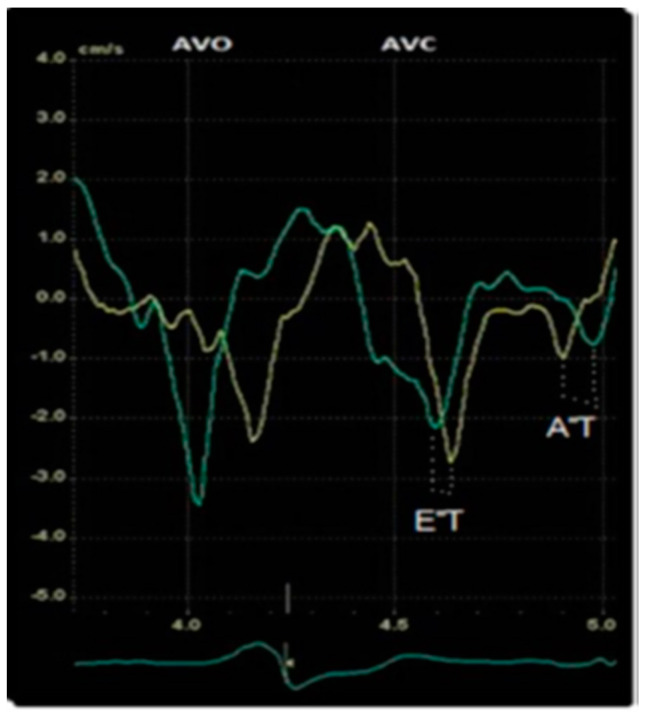
The method used to measure E″T and A″T via offline TDI timing assessment. Green line = lateral velocity curve; yellow line = septal velocity. AVO = aortic valve opening, AVC = aortic valve closure.

The assessment of diastolic dyssynchrony was conducted during the diastolic phase using offline Q-Analysis function with cTDI timing to determine the time intervals between the E″ and A″ peaks in the basal septal and lateral walls (Figure 2). Both parameters were assessed before and after CRT, outside the ejection phase.

In addition, the following parameters underwent assessment: left-ventricular ejection fraction (LVEF), left-ventricular end-diastolic and end-systolic diameters (LVEDD and LVESD), left-ventricular end-diastolic and end-systolic volumes (LVEDV and LVESV), ratio of peak flow velocity in early diastole and peak flow velocity in late diastole during atrial contraction (E/A), E/E′ ratio, and measurements of left atrial dimensions, area, and volume (LAd, LAa, and LAv).

### 2.4. Device Programming and Exercise Test

Cardiac device assessments were conducted post-implantation, 24 h later, and during all subsequent follow-up visits. An ECG pacing on/off was recorded while configuring all pacemakers with a base heart rate of 60 bpm and a maximum tracking rate (MTR) of 130 bpm. Device interrogation primarily focused on fusion pacing quality and quantity (LV threshold, percentage of ventricular pacing, and AV interval). Individually programmed AV intervals were optimized, drawing upon substantial clinical trials that have demonstrated the clinical advantages of CRT, such as MIRACLE [7] and CARE-HF [8], to achieve the best fusion capture and maximize diastolic filling time through PW-Doppler echocardiography. All patients achieved a left-ventricular (LV) fusion pacing percentage exceeding 98%.

The exercise test followed the Bruce protocol, with a 25-watt workload increase every 2 min. During ET, all patients underwent beat-to-beat ECG analysis. For those with atrial fibrillation, the ET was conducted after converting to sinus rhythm.

**Figure 3:**
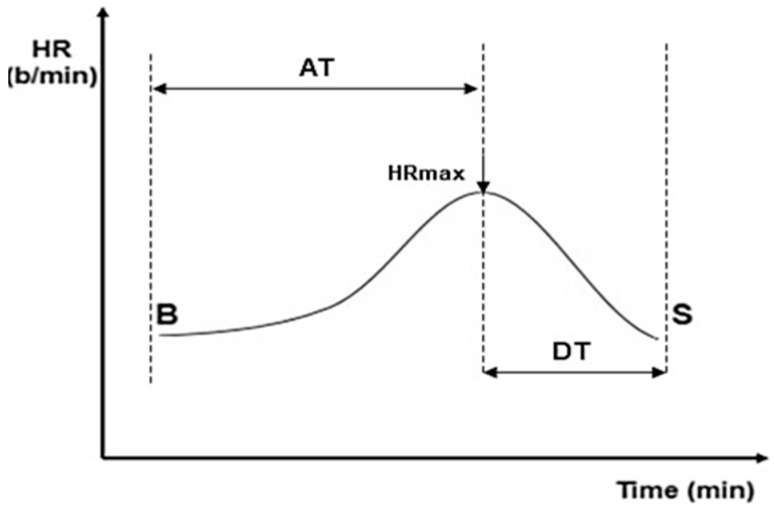
Graphic representation of the heart rate recovery index (HRRI = AT/DT). B = begin exercise; S = stop exercise; HR = heart rate; HRmax = maximum heart rate; AT = acceleration time; DT = deceleration time (adapted with permission from Cozlac et al. [6]).

An important consideration in our study is the interindividual variability in exercise performance, which may have influenced the HRRI measurements. Despite utilizing the Bruce protocol, patient responses to exercise can differ markedly due to variations in baseline physical conditioning, the presence of comorbidities, and other uncontrolled factors. This variability introduces a potential limitation regarding the reproducibility and consistency of HRRI values. The CRT evaluation during ET encompassed minimum and maximum heart rate, acceleration time (AT), deceleration time (DT), and the AT/DT ratio denoted as the heart rate recovery index (Figure 3).

To ensure optimal fusion pacing, various interventions were employed, involving adjustments to the MTR and dynamic atrioventricular interval. Additionally, beta blocker or ivabradine dosages were fine-tuned to stabilize the PR spontaneous interval [9]. Rate modulation response programming addressed cases of chronotropic incompetence.

### 2.5. CRT Response

The response assessment to CRT was predicated upon a predefined set of criteria [10]: clinical measures denoted by enhancements in NYHA functional class, quality of life, ET duration, and workload; and echocardiographic indicators of LV reverse remodeling, characterized by a greater than 5% elevation in LVEF, a 15% reduction in LV end-systolic/diastolic volume, and decreased mitral regurgitation degree. Lastly, the criteria involved outcome measures, represented by a decrease in heart failure-related hospitalizations, reduced morbidity, and a decline in all-cause mortality.

To maintain constant fusion and optimize the response to CRT, exercise tests, device programming, and medication adjustments were implemented during each 6-month follow-up.

Patients were divided into three groups: super-responders (SR), responders (R), and non-responders (NR). SR patients were defined as those with LVESV/LVEDV improvement ≥ 30% and shorter E″T/A″T and DT, while the responders were those with 15% decrease in LVES/DV. The non-responder group was characterized by a reduction in LVESV/DV < 15%, larger E″T/A″T, and prolonged DT.

### 2.6. Statistical Analysis

Continuous variables were presented as mean values along with their corresponding standard deviations, while categorical variables were expressed as proportions. The analysis of categorical variables involved the use of a chi-square test, and for continuous variables, a *t*-test was employed. Receiver operating curve (ROC) analysis was conducted to identify the optimal threshold values for E″T and A″T. Variables exhibiting a *p*-value below 0.05 were regarded as statistically significant. Statistical analysis was performed using IBM SPSS Statistics 23 Software (IBM Corp., Armonk, NY, USA). Plotting was executed using Python (version 3.6.3, Python Software Foundation, Wilmington, DE, USA) with the Matplotlib (version 1.5.3) package for data visualization.

## 3. Results

### 3.1. Cohort Baseline Demographics

The analytical group comprised seventy-two patients initially implanted with an RA/LV DDD pacing system. Notably, two patients required the implantation of a triple-chamber CRT-P system due to acute AV block complications during the cardiac implant procedure. Among the initially assessed 72 patients, 5 were excluded from the study due to the inability to perform strain rate analysis, mainly attributed to poor echocardiographic image quality. Additionally, the effort test was not feasible for three patients, given their orthopedic conditions.

The final true RA/LV CRT-P group consisted of 62 patients. The LV lead position was posterior in 8 patients (13%), postero-lateral in 24 patients (38%), lateral in 20 patients (32%), and anterolateral in 7 patients (11%); epicardial leads were needed in 3 patients (6%).

All patients met the Strauss LBBB criteria [11] and the baseline ECG data showed an average QRS interval of 164 ± 18 ms and a QRS axis of −23 (±37) degrees. Over 75% of patients received maximum pharmacological treatment upon admission, based on clinical and paraclinical variables. A limited proportion of patients received sacubitril/valsartan as part of their baseline treatment. This observation can be attributed to the fact that 35 patients underwent implantation prior to 2017. During the follow-up period, these medications were introduced, alongside SGLT2 inhibitors, in accordance with the latest ESC guidelines. A comparison between the super-responder, responder, and non-responder groups regarding the main demographic parameters, associated pathology, and medical treatments is shown in Table 1.

### 3.2. Follow-Up Outcomes

The mean follow-up was 45 ± 19 months. After 6 months of treatment, out of the 62 patients, 34% were classified as super-responders, 61% as responders, and 5% as non-responders. The average EF at baseline was 27 ± 5.2%. Diastolic dysfunction (DD) type III was identified in 29% of the patients, 63% had type II DD, and 8% were diagnosed with type I DD. Severe mitral regurgitation (MR) at baseline was found in 12% of patients, 32% of patients had moderate MR, and 56% had mild MR.

During the final follow-up, severe MR was exclusively present among non-responders. LA reverse remodeling was evident in both super-responders and responders, and every patient demonstrated a significant improvement in pulmonary artery systolic pressure (PASP). A comparison between the baseline and the follow-up echocardiographic data is presented in Table 2.

The main comparative data for ET and dyssyncrony parameters are presented in Table 3.

As noted earlier, each follow-up visit included not only echocardiography but also an exercise test, adjustments to device programming, and modifications to medical treatment. Following the exercise test, 40% of patients received device optimization, which involved increasing MTR to 145 beats per minute and reprogramming the dynamic AV interval. Furthermore, beta blocker (BB) and ivabradine titration were implemented in more than 100 cases to maintain and stabilize the AV interval.

**Figure 4:**
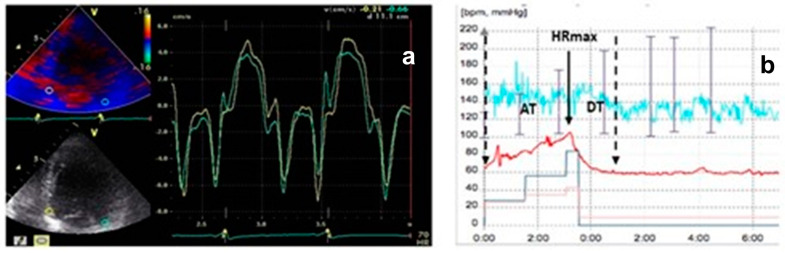
(**a**) Super-responders’ TDI pattern; green line = lateral velocity curve; yellow line = septal velocity curve. (**b**) HRRI diagram in super-responders; red line = heart rate.

In super-responders, a substantial LV reverse remodeling was observed (LVEDV 193.7 ± 81 vs. 243.2 ± 82 mL at baseline; *p* < 0.0028), along with reduced LV filling pressures (E/E′ 13.2 ± 4.6 vs. 11.4 ± 4.5; *p* = 0.0295). These changes correlated with improvements in the resynchronization parameters we studied. The E″T decreased from 90 ± 20 ms to 25 ± 10 ms and was associated with a shorter deceleration time during ET compared to NR (109 ± 68 ms vs. 330 ± 30 ms; *p* < 0.0001) (Figure 4).

Responders exhibited a higher heart rate recovery index (2.87 ± 1.47 vs. 0.98 ± 0.08; *p* = 0.03) compared to non-responders, along with a 65% improvement in diastolic dysfunction profile. Additionally, there was a significant decrease in E″T and A″T from 76 ± 13 ms to 51 ± 11 ms (*p* < 0.0001).

**Figure 5:**
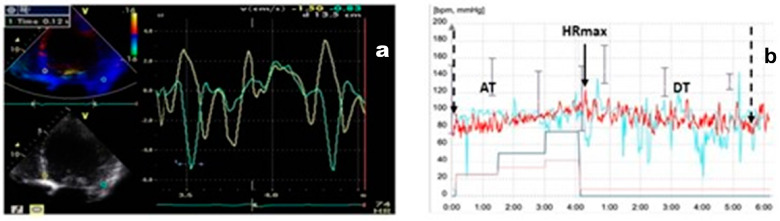
(**a**) Non-responders’ TDI pattern; green line = lateral velocity curve, yellow line = septal velocity curve (**b**) HRRI diagram in non-responders; red line = heart rate.

The NR group displayed larger baseline E″T and A″T and lower HRRI, with no statistically significant differences noted in these patients’ CRT (Figure 5).

All NR patients experienced readmission due to worsening heart failure, with a median readmission rate of 3 per year. In contrast, in R and SR groups, only six patients (10%) required hospitalization. The primary reasons for heart failure decompensation included noncompliance with dietary and medication regimens, supraventricular tachyarrhythmias, and pulmonary infections.

Non-sudden cardiac death occurred in three patients (2%) with type III DD and larger E″T/A″T (E″T > 85 ms; A″T > 30 ms) due to refractory heart failure associated with pulmonary sepsis. No deaths were recorded in the SR and R groups.

### 3.3. Predicting CRT Response

**Figure 6:**
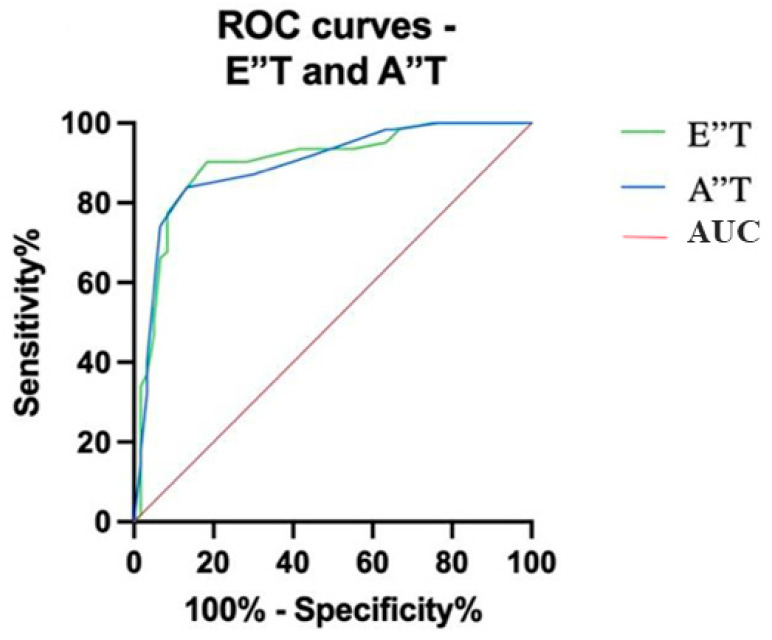
ROC (receiver operating characteristic) for E″T and A″T parameters.

In the context of CRT response prediction, our ROC analysis highlighted E″T values exceeding 80 ms (AUC = 0.8989; 95% CI 0.8401 to 0.9577; *p* < 0.0001) and A″T values greater than 30 ms (AUC = 0.8938; 95% CI 0.8351 to 0.9526; *p* < 0.0001) as robust indicators (Figure 6).

**Figure 7:**
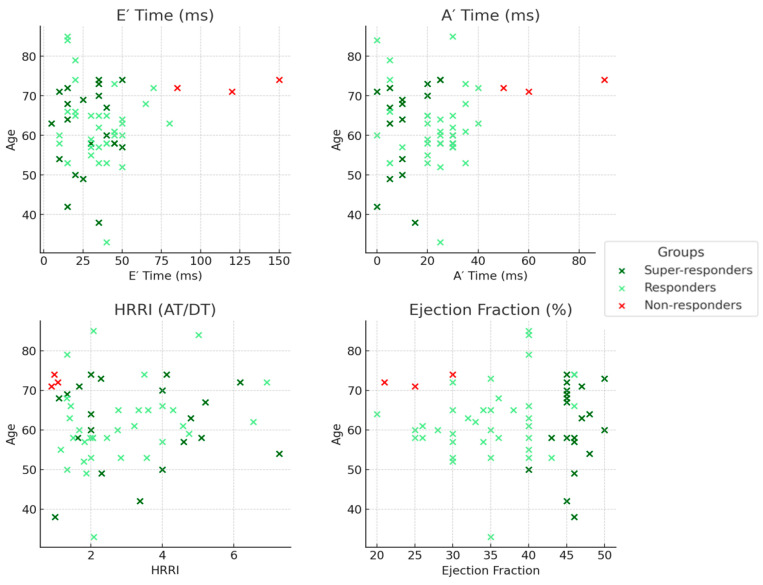
The distribution of SR/R/NR in relation to diastolic dyssynchrony/HRRI and EF.

Prolonged DT was associated with an accentuated LV dyssynchrony and nonoptimal response to CRT. Higher HHRI values (cutoff > 1.5) were observed in both super-responders and responders and were associated with significant improvement in diastolic dyssynchrony (Figure 7).

In our analysis, we elected to utilize age as a proxy for artificial jitter in the scatter plots for several significant reasons. Firstly, age is a clinically relevant variable that provides substantial context, facilitating the examination of potential associations between patient age and critical clinical parameters (E″ Time, A′ Time, HRRI, and ejection fraction). This strategy enhances data interpretability by substituting random variability with a biologically meaningful factor, thereby yielding greater insights into the interactions between age and the response categories (super-responders, responders, and non-responders). Moreover, age may function as a potential confounder or modifier in these associations, highlighting the importance of visualizing its impact on patient outcomes.

## 4. Discussion

Several studies have demonstrated that left-ventricular dyssynchrony serves as a sensitive marker of myocardial dysfunction and an independent determinant of cardiac resynchronization therapy response [12,13,14]. Dyssynchrony may also elevate the risk of arrhythmias by causing abnormal myocardial deformation, which disrupts calcium homeostasis and precipitates delayed afterdepolarizations in the resting myocardium, potentially resulting in arrhythmogenic effects [15,16,17,18,19].

Echocardiographic assessments currently demonstrate the highest efficacy in evaluating dyssynchrony and predicting therapeutic response [20,21,22,23,24,25,26]. Nevertheless, standardized criteria for their application remain insufficient. Recent evidence indicates that intraventricular dyssynchrony may serve as the most reliable predictor of response [27]. Employing deformation imaging for the assessment of intraventricular dyssynchrony enables the anticipation of subsequent reverse remodeling following left-ventricular restoration surgery or cardiac resynchronization therapy [28,29,30].

Our study emphasizes the significance of assessing diastolic asynchrony and introduces two novel echocardiographic parameters, measured both before and after resynchronization therapy, to identify new predictors of CRT response. Additionally, our research focuses on a CRT population with fusion pacing, eliminating the potential for added asynchrony from right-ventricular pacing.

To ensure consistent fusion within our patient cohort, we performed atrioventricular optimization through the application of pulsed-wave Doppler echocardiography to evaluate the mitral inflow pattern. This process involved the optimization of pharmacological regimens and the reprogramming of cardiac resynchronization therapy during follow-up assessments, which incorporated exercise testing.

In the majority of super-responders and responders, we observed a significant reduction in E″T and A″T post-implantation, suggesting the potential for a time-domain quantitative measurement to predict the LV functional recovery. Furthermore, we noticed that an extended diastolic filling time correlated with improved diastolic synchronization, resulting in reduced E″T and A″T. This underscores the significance of E″T and A″T as indirect indicators of systolic asynchrony.

ET serves as a crucial tool to enhance the response to CRT [4], with its efficacy and safety in HF patients established in prior research [31,32]. The assessment of HRRI during ET has been introduced to explore the relationship between recovery rates post-peak exertion and overall morbidity and mortality. Juven et al. found that patients exhibiting an HRR of less than 25 beats per minute faced a 2.1-fold increased risk of sudden death and a 1.3-fold increased risk of all-cause mortality [33]. Many researchers have preferred a straightforward method for calculating HRR, which involves analyzing heart rate changes from peak exercise to the first or second minute of recovery [34,35,36,37,38]. However, the methodology for HRR can be intricate and time-consuming.

To address this, we streamlined the analysis of the ET–heart rate curve by introducing HRRI, defined as the ratio of acceleration time to deceleration time. This innovative ET parameter has been shown to predict the likelihood of a positive CRT response [6]. Our findings indicated that responders and super-responders displayed a significantly steeper HRR, higher HRRI values, and improved profiles of diastolic dysfunction. Additionally, these patients exhibited shorter deceleration times during ET. The patterns observed were associated with enhancements in the dyssynchrony parameters, including a notable reduction in E″T and A″T.

Previous studies may have overlooked HRRI due to a historical focus on more well-established markers, such as EF, QRS duration, and ventricular volumes, which are easier to measure and have clearer associations with CRT response. Furthermore, the complexity of exercise testing and the variability in patient performance may have discouraged the widespread use of HRRI as a predictive marker. However, our findings suggest that HRRI provides valuable insights into autonomic function and exercise capacity, which are important predictors of CRT response and warrant further investigation in future studies.

We also need to consider the potential impact of post-implantation contrast-induced nephropathy as a potential factor in determining the CRT response [39]. This is explained by the intimate and reciprocal relationship that exists between renal and cardiac functions: renal injury can also cause a loss in cardiac performance, and a fall in cardiac output can affect renal function. Nonetheless, our study group had a comparatively low incidence of contrast-induced nephropathy and did not require significant nephrological treatments.

Other studies have corroborated these findings, showing improvements in clinical and echocardiographic parameters in HF patients at six months following CRT [7,8]. Furthermore, Thomas et al. emphasized the predictive value of heart rate deceleration in CRT response, which correlated with both functional and echocardiographic outcomes [40]. In contrast, the non-responder group demonstrated a lower HRRI, which was associated with larger baseline E″T and A″T values, and these relationships remained consistent before and after CRT. The findings highlight the potential of HRRI values as indicators of CRT response and their association with improved diastolic function and dyssynchrony reduction.

## 5. Limitations

This study has several limitations. Firstly, it was a non-randomized, single-center study, which may limit the generalizability of the findings. A randomized design with a larger, multi-center cohort is needed to validate these results and improve their applicability to broader populations. Additionally, while the study focused on fusion pacing with CRT, the absence of a fully standardized exercise testing protocol across all patients introduces potential variability in heart rate recovery index (HRRI) measurements. The small sample size and homogeneous patient population also limit the generalizability of our findings. Future studies should incorporate a standardized exercise protocol and a more diverse patient cohort to confirm the role of HRRI as a reliable predictor of CRT outcomes, in terms of improved classical CRT parameters but also correcting the diastolic dyssynchronism.

## 6. Conclusions

In conclusion, by incorporating HRRI and diastolic dyssynchrony parameters into the follow-up of CRT patients, we can improve the optimization of cardiac devices and enhance treatment strategies. Future prospective randomized trials are essential to further elucidate the long-term advantages of this integrated follow-up approach, thereby advancing the management of CRT and improving patient outcomes.

## Figures and Tables

**Table 1:** The main demographic parameters and medical treatments in all patients and subgroups.

		All Patients (N = 62)	SR (N = 21)	R (N = 38)	NR (N = 3)
Age, years (mean ± SD)		62 ± 11	61 ± 9	69 ± 10	73 ± 11
Male gender (%)		38 (61%)	11 (52%)	25 (65%)	2 (66%)
NYHA functional class, n (%)	II	6 (10%)	2 (10%)	4 (11%)	0
	III	47 (76%)	18 (86%)	27(71%)	2 (67%)
	IV	9 (1%)	1 (5%)	7 (18%)	1 (33%)
Hypertension, n (%)		35 (56%)	11 (52%)	21(55%)	3 (100%)
Diabetes mellitus, n (%)		25 (40%)	9 (43%)	14 (37%)	2 (67%)
Dyslipidemia, n (%)		48 (77%)	19 (90%)	26 (68%)	3 (100%)
Obesity, n (%)		26(42%)	10 (47%)	14 (36%)	2 (67%)
Chronic kidney disease, n (%) **		25 (40%)	5 (23%)	17 (44%)	3 (100%)
Medication	Beta blockers, n (%)	46 (74%)	18 (85%)	26 (68%)	2 (67%)
	Ivabradine, n (%)	26 (42%)	9 (43%)	15 (39%)	2 (67%)
	ACEI/ARB, n (%)	54 (87%)	19 (90%)	33 (86%)	2 (67%)
	Diuretics, n (%)	56 (90%)	18 (85%)	35 (92%)	3 (100%)
	Mineralocorticoid receptor blockers, n (%)	47 (76%)	14 (66%)	31 (81%)	2 (67%)
	Sacubitril/valsartan, n (%)	8 (12%)	2 (9%)	5 (13%)	1 (33%)

ACEI = angiotensin-converting-enzyme inhibitors; ARB = angiotensin receptor blockers; n = number; SD = standard deviation. ** Chronic kidney disease is defined as a reduction in creatinine clearance < 90 mL/min. None of the patients in our cohort had creatinine clearance < 30 mL/min.

**Table 2:** Conventional and tissue Doppler echocardiographic parameters of super-responders, responders, and non-responders at baseline and at 6-month follow-up.

	Super-Responders		*p*-Value	Responders		*p*-Value	Non-Responders		*p*-Value
	(N = 21)			(N = 38)			(N = 3)		
	Baseline	FU		Baseline	FU		Baseline	FU	
EF (%)	31 ± 4	45 ± 4	<0.0001	26 ± 5	34 ± 6	<0.0001	24 ± 4	26 ± 5	0.61
LV EDV (mL)	243 ± 82	193 ± 81	0.0028	272 ± 72	217 ± 64	0.0007	308 ± 105	278 ± 93	0.72
LV ESV (mL)	126 ± 44	75 ± 21	<0.0001	206 ± 62	148 ± 51	<0.0001	224 ± 73	174 ± 29	0.33
LA Volume (mL)	92 ± 30	89 ± 30	0.74	116 ± 40	101 ± 33	0.07	148 ± 33	141 ± 0.5	0.73
LA Surface (cm^2^)	24 ± 4	22 ± 5	0.16	30 ± 7	27 ± 7	0.06	32 ± 2	33 ± 3	0.70
PASP (mmHg)	37 ± 12	31 ± 8	0.06	42 ± 13	34 ± 10	0.0036	58 ± 6	38 ± 6	0.0151
E/E′	13 ± 4	11 ± 4	0.0295	21 ± 9	14 ± 4	<0.0001	29 ± 4	23 ± 7	0.26
E/A	2 ± 0	1 ± 0	-	1.4 ± 5	0.8 ± 3.9	0.44	2 ± 0	2 ± 0	-

All data are presented as mean ± standard deviation. EF = ejection fraction; FU = follow-up; LVEDV = left-ventricular end-diastolic volume; LVESV = left-ventricular end-systolic volume; LA = left atrium; PASP = pulmonary artery systolic pressure.

**Table 3:** Comparison of exercise test and dyssynchrony parameters between super-responders, responders, and non-responders at baseline.

	Super-Responders	*p*-Value	Responders	*p*-Value	Non-Responders
Basal HR	67.4 + 9.3	0.95	67.5 + 9.3	0.94	67.1 + 9,4
HR max	99.6 + 20.2	0.80	99.4 + 20.1	0.81	96.5 + 21.5
DT (ms)	109 ± 68	<0.0001	161 ± 78	0.0008	330 ± 30
HRRI = AT/DT	3.23 ± 1.75	0.03	2.87 ± 1.47	0.03	0.98 ± 0.08
E″ Time (ms)	90 ± 20	0.028	76 ± 13	<0.0001	118 ± 10
A″ Time (ms)	16 ± 7	<0.0001	26 ± 8	<0.0001	67 ± 9

All data are presented as mean ± standard deviation. HR = heart rate; HR max = maximum heart rate; HRRI = heart rate recovery index; DT = deceleration time.

## Data Availability

The data presented in this study are available on request from the corresponding author. The data are not publicly available due to ethics and data security reasons.
